# Talin 1 plays a significant role in the development of tolerogenic dendritic cells, which may contribute to the progression of multiple sclerosis

**DOI:** 10.1016/j.neurot.2025.e00731

**Published:** 2025-09-09

**Authors:** Kouichi Ito

**Affiliations:** RWJMS Institute for Neurological Therapeutics and Department of Neurology, Rutgers-Robert Wood Johnson Medical School, Piscataway NJ08854, USA

**Keywords:** Immune tolerance, Tolerogenic dendritic cells, Multiple sclerosis, Talin 1

Multiple sclerosis (MS) is an autoimmune disease that affects the central nervous system (CNS) [1]. It is believed that autoreactive T cells, which target myelin proteins, play a role in both the initiation and progression of MS. In healthy individuals, these autoreactive T cells circulate harmlessly in the body until an environmental stimulus mistakenly activates them, causing them to differentiate into pathogenic Th1 and Th17 ​cells. Once activated, these myelin-specific T cells infiltrate the CNS, where they trigger an immune response against myelin. This immune response recruits additional pathogenic macrophages and B cells to the CNS [2]. The presence of these immune cells activates microglia, leading to demyelination and loss of axons. Therefore, disease-modifying therapies (DMTs) that suppress the activation of immune cells contributing to MS progression have been used for the treatment of relapsing-remitting MS [3]. However, currently available DMTs use non-specific approaches that affect both pathogenic and beneficial immune responses, which may lead to several potential safety risks [4]. Therefore, it has been suggested that selectively suppressing only the autoreactive T cells associated with MS may be a better strategy, as it could reduce the adverse risks linked to non-specific immune suppression.

Dendritic cells (DCs) are a specialized type of antigen-presenting cells that play a crucial role in autoimmune diseases. They present autoantigens to T cells, which can lead to the activation of autoreactive T cells, including those specific to myelin. On the other hand, DCs are also essential for suppressing the activation of autoreactive T cells, which is vital for maintaining immune tolerance. Under steady-state conditions, a specific type of DCs known as natural tolerogenic DCs is vital for sustaining this immune tolerance and preventing the activation of autoreactive T cells. The tolerogenic DCs interact with T cells in ways that can lead to T cell deletion, anergy (unresponsiveness), or the development of regulatory T cells. Therefore, natural tolerogenic DCs reside in lymphoid organs and play a pivotal role in preventing autoimmune diseases including MS [5]. Recent studies have shown that tolerogenic DCs can be generated *in vitro* by stimulation through certain pattern recognition receptors and the presence of specific cytokines such as transforming growth factor (TGF)-β and interleukin (IL)-10 [5,6]. These inducible tolerogenic DCs loaded with myelin antigens have been successfully used to suppress myelin-specific T cells and exhibited the beneficial therapeutic effect on experimental autoimmune encephalomyelitis (EAE), an animal model of MS [[7], [8], [9], [10], [11]].

Talin-1 is an essential adaptor molecule involved in integrin signaling, which is necessary for cell adhesion and migration mediated by integrins [12,13]. Additionally, Talin-1 plays a significant role in the activation and maturation of dendritic cells (DCs). It does this by facilitating the formation of preassembled Toll-like receptor (TLR) complexes during steady-state conditions and promoting the assembly of signalosomes upon ligand binding. This process involves direct interactions between Talin-1, MyD88, and PIP5K. Talin-1 influences TLR-signaling pathways by serving as a scaffold for the myddosome in DCs, thereby impacting inflammatory responses. Notably, a deficiency in Talin-1 expression in DCs leads to a significant decrease in both the mRNA transcript and protein levels of pro-inflammatory cytokines such as IL-1β, IL-6, IL-23, and IFN-β1. This reduction in pro-inflammatory cytokines occurs through the down-regulation of NFκB, a key downstream effector in TLR signaling cascades responsible for the expression of several pro-inflammatory cytokines [14]. Therefore, reduced expression of Talin-1 in DCs is suggested to lower inflammation levels.

In this issue of Neurotherapeutics, Jia Liu and colleagues show that DCs can differentiate into tolerogenic DCs in the absence of Talin-1. In their study, bone marrow-derived dendritic cells (BMDCs) were transduced with shTalin1 lentiviral vectors, resulting in an approximately 80 ​% reduction in Talin-1 expression. These cells were used for both *in vitro* and *in vivo* experiments. The upregulation of CD80, CD86, and MHC class II induced by LPS was reduced when the expression of Talin-1 was decreased in BMDCs. Additionally, downregulation of Talin-1 suppressed the activation of the TLR4/MyD88/NF-κB signaling pathway. This was achieved by downregulating the interaction between TLR4 and IRAK4, as well as reducing the phosphorylation and ubiquitination levels of related molecules, which ultimately impaired the activation of the BMDCs. Importantly, the downregulation of Talin-1 also led to a reduction in LPS-induced expression of pro-inflammatory cytokines such as IL-1β, IL-6, and TNF-α, while increasing the expression of the anti-inflammatory cytokine IL-10. These findings suggest that the downregulation of Talin-1 predisposes BMDCs to exhibit an inactive or immature pattern of surface molecules and cytokines in response to LPS stimulation. Since immature DCs can function as tolerogenic DCs, the tolerogenic activity of Talin-1-downregulated BMDCs was examined *in vitro*. These BMDCs demonstrated a reduced capacity of CD4^+^ T cell proliferation and polarization toward Th1 and Th17 subsets. In a therapeutic experiment, MOG35-55 peptides were loaded onto Talin-1-downregulated BMDCs and administered to mice with EAE. The mice that received the treatment demonstrated an improvement in neurological symptoms and a decrease in disease-related damage. Furthermore, this treatment resulted in decreased levels of Th1 and Th17 lineages while simultaneously increasing the abundance of regulatory T cells. This study suggests that the expression of Talin-1 in DCs contributes to the development of tolerogenic DCs, which may influence the initiation and progression of MS. Notably, this study also showed that the expression of Talin-1 was found to increase during the development of EAE. Another study on MS revealed that serum levels of soluble Talin-1 were significantly higher in comparison to healthy controls, with elevated concentrations observed during relapse as opposed to remission [15]. These studies indicate that Talin-1 in DCs could be a promising therapeutic target for immune dysregulation in multiple sclerosis (MS). Additionally, Talin-1-deficient DCs could function as tolerogenic DCs to promote immune tolerance. In clinical studies for MS using tolerogenic DCs, it is crucial to identify the tolerogenic epitopes. Initially, T cells target a specific epitope of myelin proteins; however, they later expand their recognition to include additional myelin-associated epitopes. This phenomenon, known as epitope spreading, contributes to disease progression and is associated with increased severity and chronicity of the condition [16]. Consequently, designing a specific myelin epitope for tolerogenic DC therapy can be quite challenging. Therefore, further research is necessary to identify tolerogenic antigens in MS for this treatment.

## Author contribution

Kouichi Ito: Writing – review & editing.

## Declaration of competing interest

The authors declare that they have no known competing financial interests or personal relationships that could have appeared to influence the work reported in this paper.
